# Peritonitis following duodenal injury due to seatbelt trauma: A case manifesting immediately post-surgery for thoracolumbar fracture-dislocation

**DOI:** 10.1016/j.ijscr.2025.110829

**Published:** 2025-01-03

**Authors:** Hisashi Serikyaku, Shoichiro Higa

**Affiliations:** Department of Orthopedic Surgery, Naha City Hospital, Okinawa, Japan

**Keywords:** Seat belt injury, Thoracolumbar fracture dislocation, Hollow visceral injury, Peritonitis

## Abstract

**Introduction:**

Although abdominal organ damage due to motor vehicle accident is often evident immediately after the injury and urgent operation is performed, it has been reported that minor injuries such as hollow viscus may become apparent during the course of treatment and require urgent surgery.

**Case report:**

The Authors present the case of a 42-year-old female who developed peritonitis immediately after undergoing surgery for thoracolumbar fracture-dislocation caused by a traffic accident. The patient exhibited no abdominal symptoms, such as nausea, vomiting, or abdominal wall rigidity, and had no difficulty with oral intake preoperatively. The patient was consulted to an abdominal surgeon, who proceeded with an emergency surgery. Intraoperatively, the duodenal injury was identified and meticulously repaired. Postoperatively, the patient was transferred to the intensive care unit for ongoing critical care management. By postoperative day 60, the patient was able to walk independently and was discharged.

**Discussion:**

Preoperative diagnosis of patients with delayed bowel obstruction due to seat belt injuries poses a challenging task for surgeons. The diagnosis and treatment of isolated duodenal injuries has been reported to be difficult because of the retroperitoneal organ. In the clinical management of seatbelt injuries, attention should not be exclusively directed toward the more conspicuous spinal fractures, but the possibility of concomitant bowel injuries must also be carefully considered.

**Conclusion:**

Treatment should be carried out with the awareness that intestinal injuries may be present, even in the absence of abdominal symptoms during the initial examination.

## Highlights

• We summarize the clinical manifestations and treatment of a case of postoperative peritonitis following thoracolumbar fracture dislocation caused by the seat belt in a traffic accident.
• Peritonitis developed shortly after reduction and fixation surgery for thoracolumbar fracture-dislocation, with contrast-enhanced CT revealing a suspected duodenal injury and free gas around the liver.
• Following abdominal surgery, the peritonitis improved, and with subsequent rehabilitation, the patient regained the independent ambulation.
• In cases of thoracolumbar fracture-dislocation caused by seat belt injuries, it is prudent to consider the possibility of hollow visceral injuries during medical treatment.

## 1 Introduction

While seat belt use has significantly reduced the risk of mortality, concerns remain regarding thoracolumbar spine injuries and abdominal organ damage directly associated with seat belts themselves. Small intestinal injuries occurred in 5–15 % patients with the blunt abdominal trauma including motor vehicle accidents [1]. Although abdominal organ damage due to motor vehicle accident is often evident immediately after the injury and urgent operation is performed, it has been reported that minor injuries to abdominal organs may worsen during the course of treatment and require urgent surgery [2].

We report a case of peritonitis that developed immediately after surgery performed two days post-injury for a thoracolumbar dislocation fracture caused by a seatbelt injury.

## 2 Case report

**Fig. 1:**
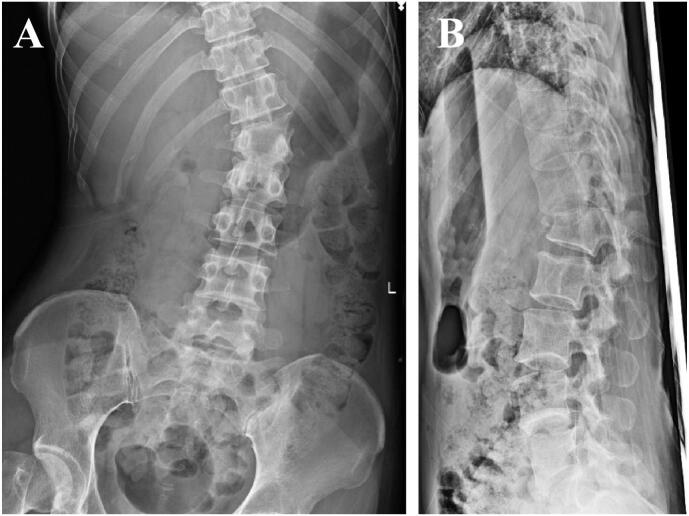
Simple roentgenograms revealed a dislocated fracture of Th12/L1 and fractures of the right 10th–12th ribs (A, B).

**Fig. 2:**
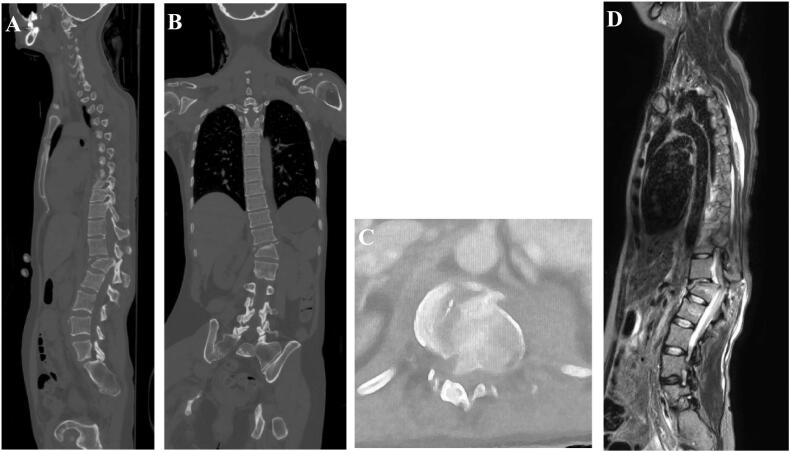
The sagittal, coronal reconstructions, and axial CT images demonstrated a fracture-dislocation at the Th12/L1 level (A–C). The MRI T2-weighted images of the whole spine revealed a fracture-dislocation at the Th12/L1 level, with significant compression of the dura mater (D).

A 42-year-old female passenger in the front seat was transferred to a nearby emergency hospital after her car collided with a wall while driving. She was conscious and her vital signs were stable. Severe lumbar back pain and complete paraplegia were observed, with a loose anal sphincter and absence of voluntary urination. Simple roentgenograms revealed a dislocated fracture of Th12/L1 and fractures of the right 10th–12th ribs (Fig. 1). Contrast-enhanced computed tomography (CT) revealed Arbeitsgemeinschaft für Osteosynthesefragen (AO) classification [3] type C fracture-dislocation and suspected transverse mesenteric injury. Magnetic resonance imaging (MRI) also revealed a similar fracture-dislocation, with findings indicating significant compression of the dura mater (Fig. 2).

The patient was urgently airlifted to our hospital via helicopter for the treatment of a fracture dislocation at the Th12/L1 level. The patient was fully conscious on arrival at our hospital. Complete paraplegia was observed, with minimal pain in the lower limbs. The anal sphincter was non-functional, and there was no intra-anal sensation (ASIA Grade B). The abdomen was observed to be flat and soft, with normal bowel sounds auscultated. The patient had a thoracolumbar spine injury classification score of 13 in the AO classification. Surgery was scheduled for two days after the date of injury. In the meantime, the patient was kept on bed rest and was allowed to eat, but she had no problems after eating.

**Fig. 3:**
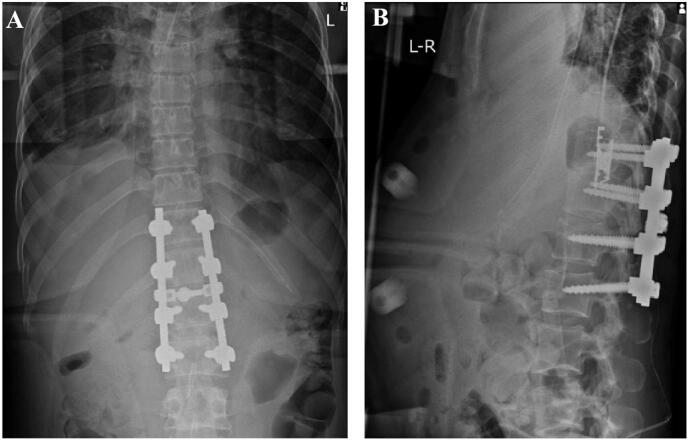
The postoperative X-ray images demonstrated good reduction on both anteroposterior (A) and lateral views (B).

**Fig. 4:**
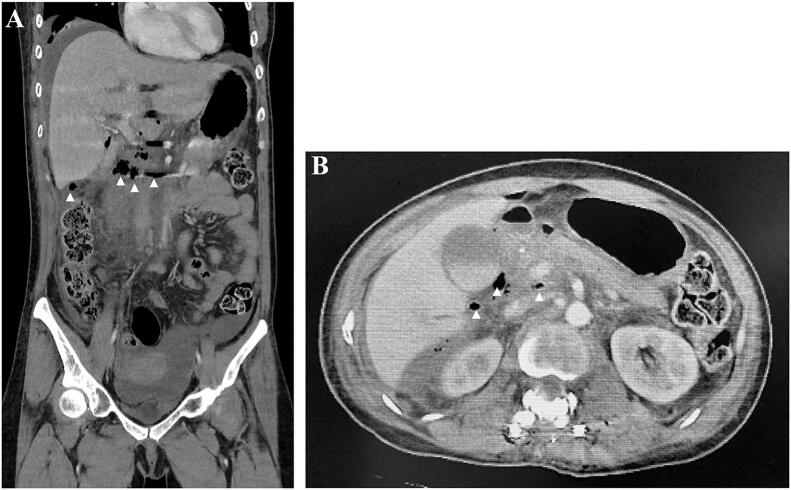
The contrast-enhanced chest and abdominal CT scans revealed free air surrounding the duodenum and liver, along with fat stranding and fluid accumulation (arrow heads) (A, B).

**Fig. 5:**
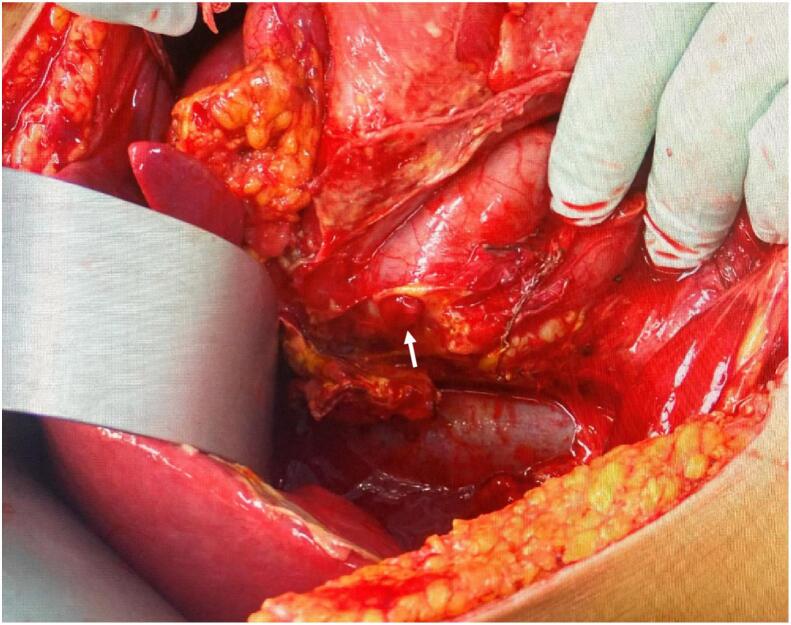
A 1 cm perforation was revealed on the dorsal aspect of the descending duodenum without any accompanying injury to the pancreas (arrow).

The operation was performed under general anaesthesia in the supine position on an operating table. A median skin incision was made and the paraspinal muscles from the Th10 spinous process to the L3 spinous process were dissected, exposing the spinous processes and lamina, as well as the transverse processes. The paraspinal muscles were bleeding and damaged. The operation was performed under a microscope thereafter. The right inferior articular process of Th12 deviated from the right superior articular process of L1 and was displaced ventrally. The caudal aspect of the right inferior articular process of Th12 was resected, and the dislocation was meticulously repaired. Fortunately, there was no evidence of dural tear and no spinal fluid leakage. The pedicle screws were placed from the Th11 to L2 on both sides, and after connecting the rods, the dorsal aspect of lamina was decorticated and grafted with local bone. The wound was closed in layers, the surgery was concluded (Fig. 3), and the patient was transferred to the stretcher in the supine position. The patient's abdomen was distended and firm, and after awakening, she complained of severe abdominal pain and exhibited restless. The abdominal surgeon was consulted and contrast-enhanced CT imaging of the abdomen revealed the presence of free air extending from the duodenum to the perihepatic region, as well as fatty opacity and ascitic effusion (Fig. 4). The same CT scan demonstrated the optimal position of the pedicular screws, with no perforation of the anterior wall of the vertebral bodies. Emergency laparotomy was performed. A perforation of approximately 1 cm in the dorsal aspect of the descending part of duodenal was observed, but there was no evidence of damage to the surrounding liver or pancreas (Fig. 5). The perforation site was primary repaired and covered with the large omentum, a drain was placed and the wound closed. The patient was managed in the ICU while intubated and a thoracic drain was placed on the third postoperative day due to a pleural effusion in the right pleural cavity. The tracheal tube was removed on postoperative day 4. Oral intake of water was initiated on postoperative day 7, and the chest drain was removed and oral intake began on postoperative day 8. The abdominal drain was removed on postoperative day 10, and the patient was transferred to the general ward on postoperative day11.

Neurologically, nociception in both lower limbs was evident on postoperative day 3, with the right hip adduction strength rated as 2 on the Manual Muscle Testing (MMT) scale. On postoperative day 4, contraction of the right quadriceps muscle was observed. On postoperative day 5, right knee flexion and extension were rated as 2 on the MMT scale, and by postoperative day 7, ankle flexion and extension on the right side were observed. On postoperative day 8, muscle contraction of the left thigh adductors was observed, followed by significant improvement in muscle strength. By postoperative day 60, the patient was able to walk independently and was subsequently discharged. At the one-year postoperative follow-up, the patient required manual assistance for defecation but was able to void spontaneously, ambulate independently, and showed no sensory deficits (ASIA Grade E).

## 3 Discussion

In a study involving about 520,000 cases of spinal trauma, 36.8 % involved thoracic spine injuries, and among those, 52 % were attributed to traffic accidents. The study reported that 2 % of thoracic spinal cord injury cases were accompanied by intestinal injury [4]. The clinical presentation of seat belt syndrome is frequently non-specific, often resulting in delays in both diagnosis and therapy. The presence of abdominal ecchymosis in seatbelt injuries is reported to be evidence supporting 7-fold increase of abdominal injury [5].

Preoperative diagnosis of patients with delayed bowel obstruction due to seat belt injury is a challenging task for surgeons. Patients may present with few symptoms, stable vital signs, no clinical signs of peritonitis, and initial imaging studies may even be negative [6]. Jones reported that intra-abdominal injury following blunt trauma including road traffic accident becomes clinically apparent by 8 h 25 min after injury on all 285 cases of blunt abdominal injuries [7]. Duodenal injuries occur in 11.2–26 % of all blunt abdominal trauma, often in conjunction with other organ injuries, with isolated injuries reported to be rare [[8], [9], [10], [11]]. The diagnosis and treatment of isolated duodenal injuries has been reported to be difficult because of the retroperitoneal organ [12].

Ozabi has documented two cases of delayed diagnosis of bowel injury. The first case involved the onset of abdominal pain and tachycardia 24 h post-accident, with exploratory laparotomy revealing devascularization of the terminal ileum and mesenteric injury. The other case presented 48 h after the accident with symptoms of abdominal pain, vomiting, fever, and tachycardia, where exploratory laparotomy identified an almost circumferential rupture of the ileum [2].

In suspected cases, repeated CT scans are recommended at regular intervals over several hours. This approach can facilitate the early diagnosis of intestinal injuries [13]. It has been reported that the sensitivity and specificity of CT scans in predicting hollow viscus injury in blunt trauma were 55.33 % and 92.06 %, respectively, with positive and negative predictive values of 61.53 % and 89.23 %, respectively [14]. Among adjunctive diagnostic modalities, ultrasound proves valuable only in the context of the Focused Assessment with Sonography for Trauma (FAST) in hemodynamically unstable patients [15]. However, due to its low sensitivity, it is not a reliable tool for the detection of intestinal or mesenteric injuries [16].

On initial examination at our institution, the patient's abdomen was soft and flat, bowel sounds were normal, and no seatbelt sign was evident. Based on these findings, the potential for injuries to abdominal organs other than the mesenteric injury identified at the initial hospital was not considered during our evaluation. Given the high frequency of concurrent intestinal injuries associated with mesenteric injuries [13], sequential CT imaging should have been performed even when symptoms were not apparent. Oral intake should also have been initiated after careful assessment of the abdomen as it may have overloaded the site of the intestinal injury. The patient's postoperative abdominal symptoms may have been caused by the supine position and the reduction of the dislocated vertebrae, which resulted in stretching of the duodenum and exposure of the injured area. In the clinical management of seatbelt injuries, attention should not be exclusively directed toward the more conspicuous spinal fractures, but the possibility of concomitant bowel injuries must also be carefully considered.

The Type C injury has neurological deficit in the average of 55 % [17]. In a series of 16 cases of AO type C fracture-dislocations at the thoracolumbar junction, Feng reported the presence of dural tears and cerebrospinal fluid leakage in 11 cases. Of these, 8 cases involved dorsal injuries, while 3 cases involved ventral injuries [18]. Of the 16 cases of AO type C dislocated fractures, five cases of preoperative ASIA Grade B were reported, with three cases improving to Grade C and two cases to Grade D postoperatively. In this case, no dural injury or cerebrospinal fluid leakage was observed. Through meticulous reduction and fixation without spinal cord and dural injury, the patient, while still requiring manual assistance for defecation, recovered to ASIA Grade E [19].

## 4 Conclusion

Here, we present a case of duodenal injury caused by a seat-belt injury, which manifested immediately after surgery for thoracolumbar fracture-dislocation. Preoperative contrast-enhanced CT of the chest and abdomen for the fracture-dislocation suggested the possibility of a mild mesenteric injury; however, the patient exhibited no abdominal symptoms, such as nausea, vomiting, or abdominal wall rigidity, and had no difficulty with oral intake. It is presumed that the symptoms manifested due to pressure applied to the site of the duodenal injury during the prone position and reduction of the thoracolumbar fracture-dislocation during surgery. In cases of thoracolumbar fracture-dislocation caused by seat belt injuries, it is prudent to consider the possibility of hollow visceral injuries during medical treatment.

This study has been reported in line with the SCARE criteria [19].

## Patient consent

Written informed consent was obtained from the patient for publication of this case report and accompanying images. A copy of the written consent is available for review by the Editor-in-Chief of this journal on request.

## CRediT authorship contribution statement

Hisashi Serikyaku: operator, manuscript drafting, writing, literature search.

Shoichiro Higa: Colleague who assisted in the operation.

## Ethical approval

Ethical approval for this study (2024a24) was provided by the Ethical Committee Naha City Hospitals, Okinawa, Japan on 12 November 2024.

Sources of support that require acknowledgement: The manuscript submitted does not contain information about medical device(s)/drug(s). No funds were received in support of this work. No relevant financial activities outside the submitted work.

## Guarantor

Dr Hisashi Serikyaku.

## Sources of funding

N/A.

## Declaration of competing interest

N/A.
